# A Case of Orf Disease Complicated with Erythema Multiforme and Bullous Pemphigoid-Like Eruptions

**DOI:** 10.1155/2015/105484

**Published:** 2015-07-29

**Authors:** Shahriar Alian, Fatemeh Ahangarkani, Sara Arabsheybani

**Affiliations:** Antimicrobial Resistance Research Center, Department of Infectious Diseases, Mazandaran University of Medical Sciences, Sari, Iran

## Abstract

Parapoxvirus infection in sheep and goats is usually referred to as *contagious pustular dermatitis*/*ecthyma*, or *orf*, and the corresponding human infection is referred to as *orf*. In humans, after a brief incubation period of 3 to 5 days, lesions begin as pruritic erythematous macules and then rise to form papules, often with a target appearance. Lesions become nodular or vesicular, and orf lesions often ulcerate after 14 to 21 days. Erythema multiforme and bullous pemphigoid have been associated with parapoxvirus infections and they are rare complications of orf disease. In this case report, we presented a 36-year-old woman with history of contact with sheep, developing a typical orf lesion that is complicated with erythema multiforme and bullous pemphigoid-like eruptions.

## 1. Introduction

Parapoxviruses are common pathogens of sheep, goats, and cattle [1] and chamois [2]. Human infection, characterized by localized epithelial lesions, is an occupational hazard for those who handle infected animals. Parapoxvirus infection in sheep and goats is usually referred to as* sore mouth, scabby mouth, contagious pustular dermatitis/ecthyma*, or* orf*, and the corresponding human infection is referred to as* orf*. Taxonomically, the relevant parapoxvirus species is referred to as* Orf virus*; synonyms are contagious pustular dermatitis virus and contagious ecthyma virus.

Infection manifests as localized lesions at the site of inoculation by a diseased animal. The portal of entry is usually a break in the skin. Six stages of clinical disease are described. Milker's nodules, also known as paravaccinia and pseudovaccinia, are cutaneous lesions caused by infection with parapoxvirus of bovine origin.

After a brief incubation period of 3 to 5 days, lesions begin as pruritic erythematous macules and then rise to form papules, often with a target appearance (days 7 to 14). Lesions become nodular or vesicular, and orf lesions often ulcerate after 14 to 21 days; this ulceration has been referred to as the* acute stage*. Complete healing can take up to 4 to 6 weeks.

Erythema multiforme and bullous pemphigoid have been associated with parapoxvirus infections and they are very rare complications of orf [1].

Here we describe a case of orf disease that appeared with erythema multiforme and bullous pemphigoid-like eruptions.

## 2. Case Report

A 36-year-old woman, with no relevant medical history, presented with a necrotic ulcer on her distal part of the third finger of the left hand. She had history of trauma and ulceration of this finger about one month before admission, and 10 days after trauma she had contact with dead sheep. Three days after this contact, she presented with a small papular lesion on the distal part of the third finger of the left hand.

This lesion gradually progressed to a greater vesiculopustular lesion about 1.5 cm in diameter during 2 weeks.

**Figure 1:**
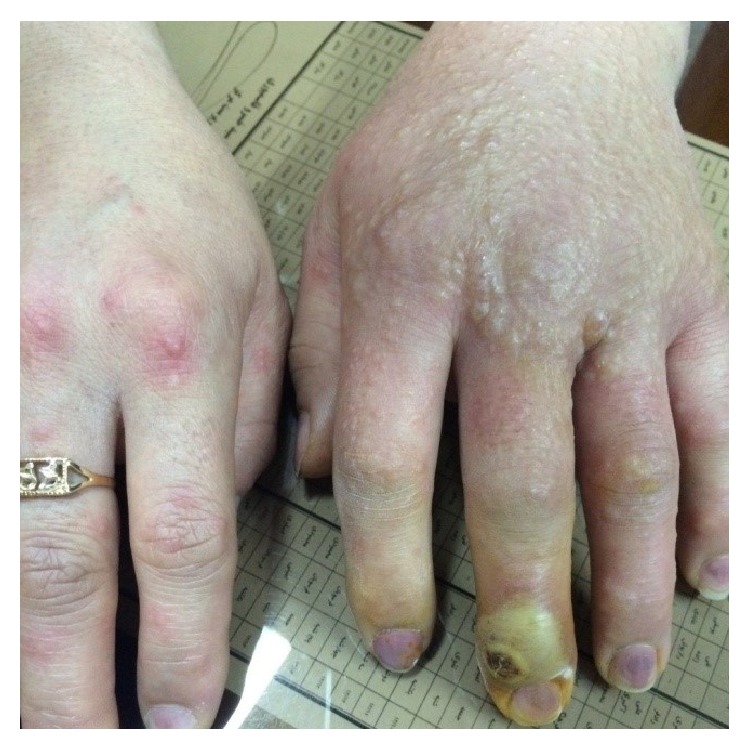
Orf lesion and papulovesicular rash one day before admission.

**Figure 2:**
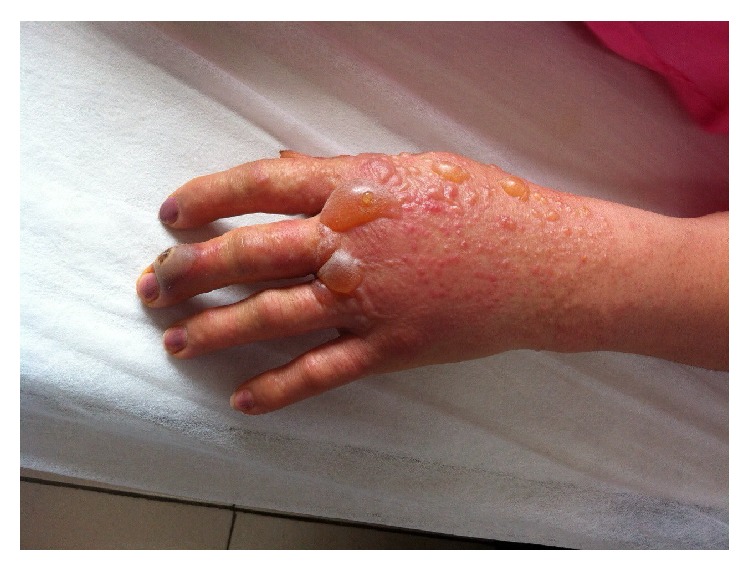
Vesiculobullous lesions, the first day of admission.

**Figure 3:**
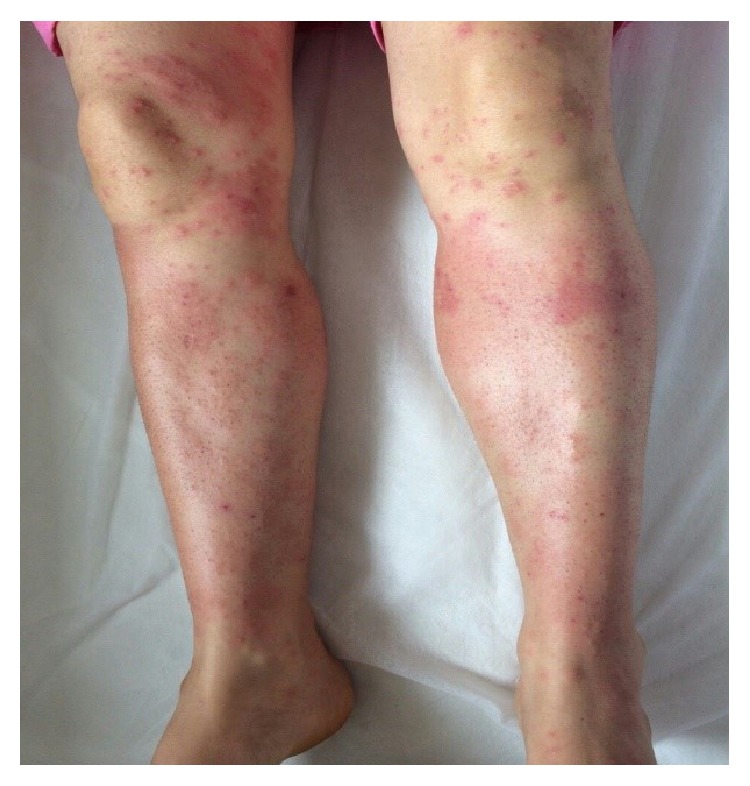
Generalized papulovesicular and bullous rash, the first day of admission.

**Figure 4:**
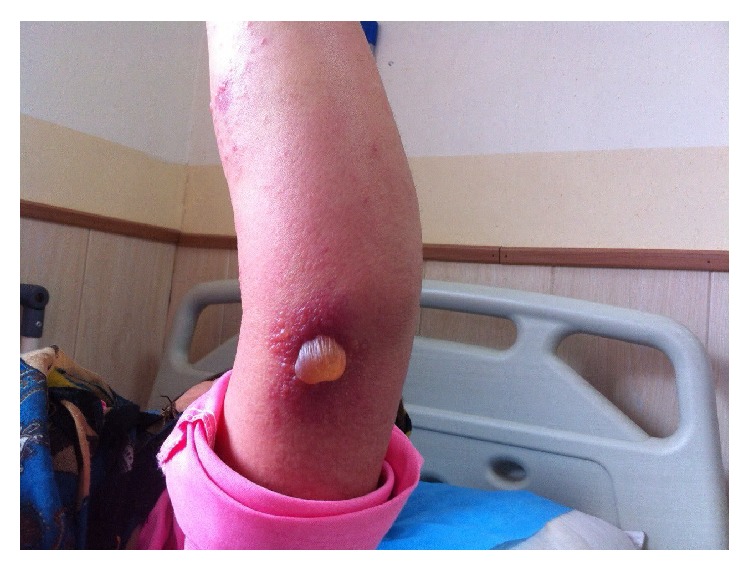
Vesiculobullous lesions on her left elbow.

She manipulated this lesion 5 days before admission. One day before admission to hospital, she presented with generalized maculopapular rashes which some of them were developed to vesicular and bullous lesions particularly on skin of left upper extremity (Figures 1, 2, 3, and 4).

The patient did not have constitutional symptoms or high fever. She suffered from itching, redness, and swelling of her hands (that was prominent in the left hand) and feet.

**Figure 5:**
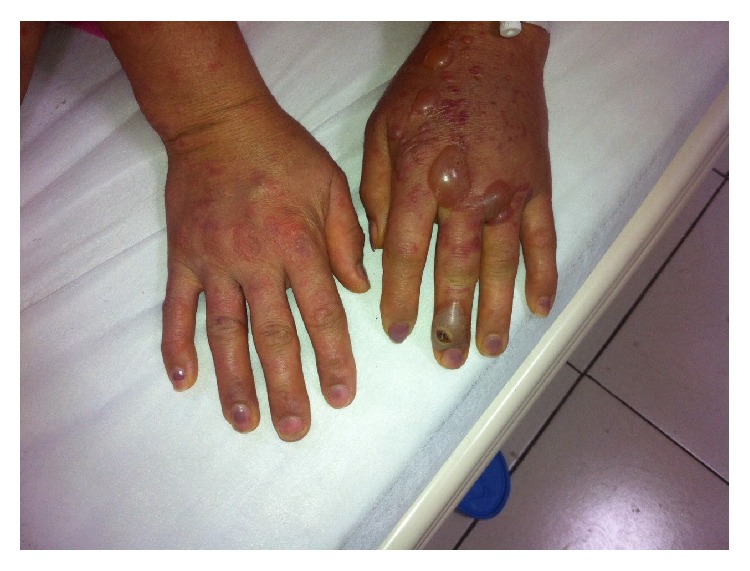
Hand lesions, 2 days after hospitalization.

**Figure 6:**
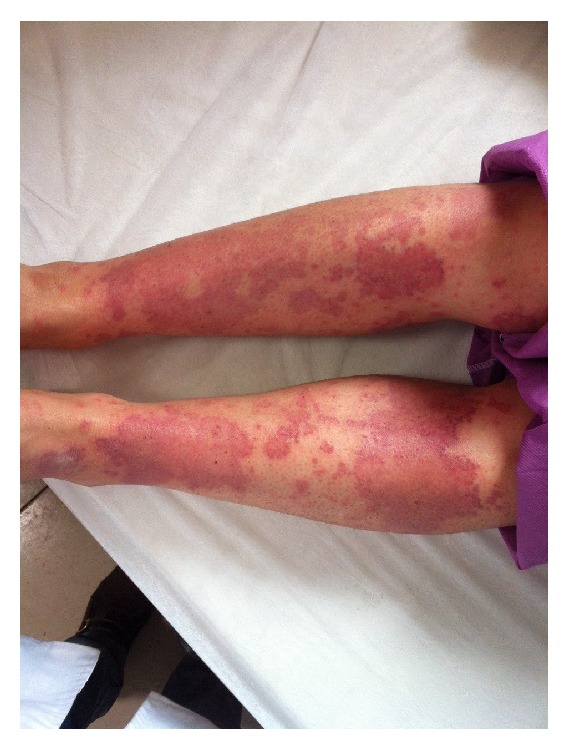
Target-shaped lesions on her lower extremities after 2 days.

**Figure 7:**
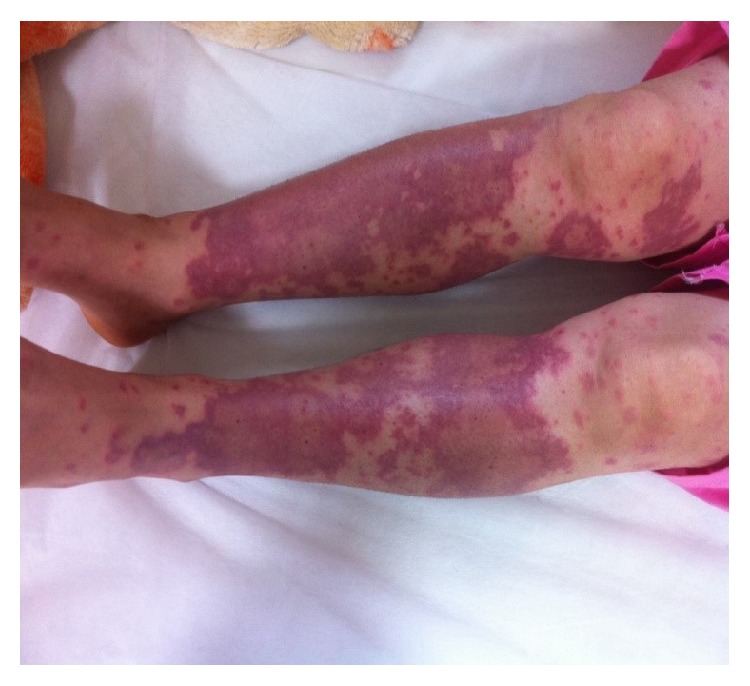
Target-shaped lesions after 3 days.

**Figure 8:**
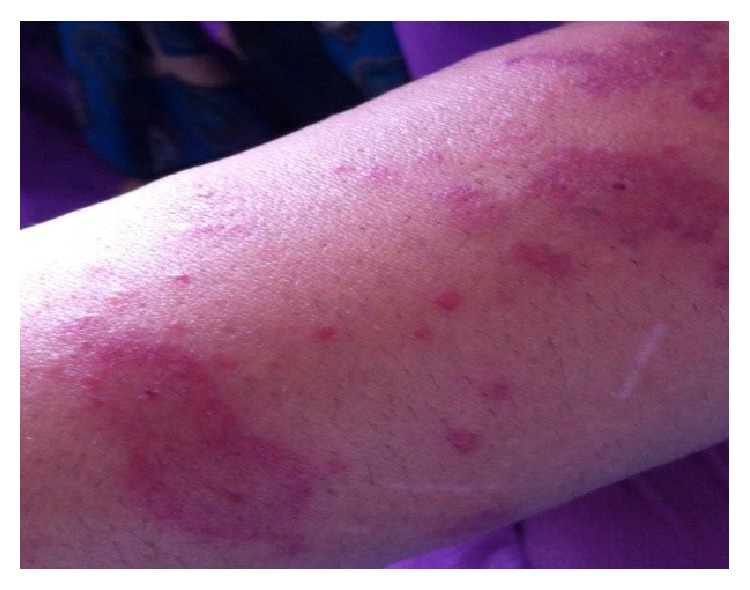
Target lesions on her arm.

On the second day of admission, multiple target-shaped lesions were initiated on her arms, legs, and trunk (Figures 5 and 6) which progressed after one day (Figures 7 and 8).

Microscopic finding of skin biopsy revealed subepidermal bulla which contains fibrin and inflammatory cells composed of eosinophils, some lymphocytes, and neutrophils. The dermis showed edema and perivascular infiltration of lymphocytes, neutrophils, and eosinophils. These findings were consistent with bullous pemphigoid.

The patient was diagnosed with orf on the basis of her typical history of contact with infected sheep and typical lesion, concomitant with erythema multiforme and bullous pemphigoid-like eruptions as complications of orf disease.

**Figure 9:**
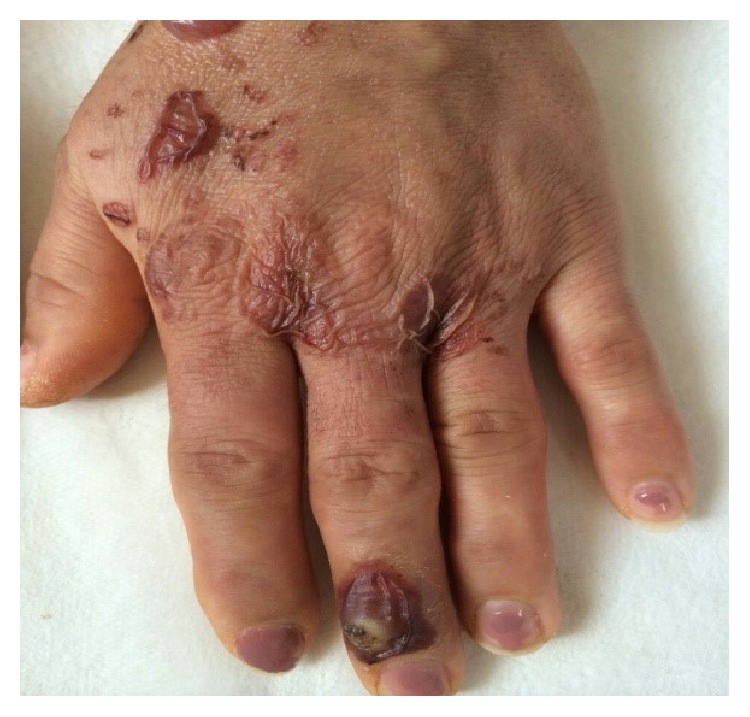
One week after hospitalization.

**Figure 10:**
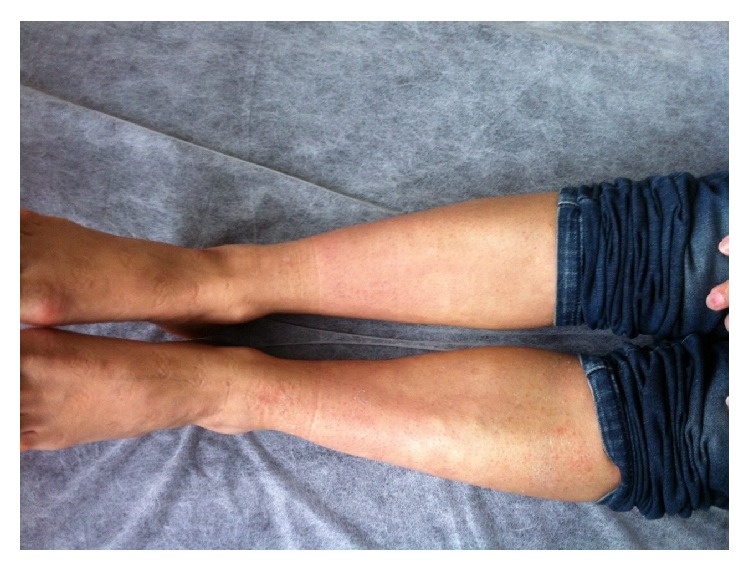
Three weeks after initial lesion.

**Figure 11:**
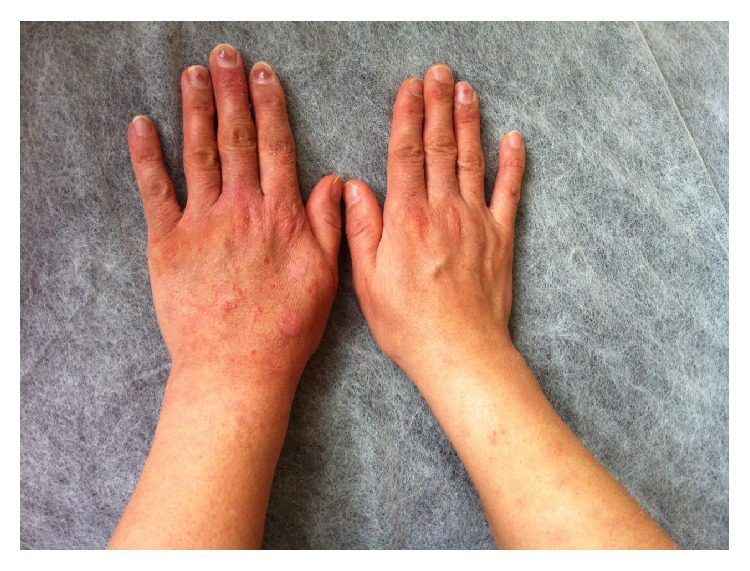
Improvement of skin lesions after 3 weeks.

She was treated with prednisolone 50 mg/day (the dose was tapered down during 2 weeks). After 5 days of treatment, the patient's lesions improved dramatically (Figures 9, 10, and 11).

Figures 1–11 show orf lesion on her 3rd finger of left hand and bullous pemphigoid lesions and target lesions on her hand, arm, and legs and her amelioration process.

## 3. Discussions

Orf virus (i.e., contagious ecthyma virus/contagious pustular dermatitis virus) is specific to sheep and goats. The majority of human parapoxvirus infections probably go unreported, because many farmers and rural physicians are aware of the disease and make a diagnosis solely on the basis of clinical findings. No human-to-human transmission of parapoxvirus infection has been reported [3].

Milkers' nodules occur most frequently on the fingers or hands of people who have had contact with an infected sheep. Following an incubation period of 3–7 days, the lesions begin as an erythematous papule, possibly with associated vesicles. This progresses over a period of weeks and forms a firm nodule that is reddish blue to brown in color. In some cases, there may also be suppuration and scabbing [3].

Differential diagnoses include pyoderma, herpetic whitlow, cowpox, pseudo cowpox (Milker's nodule), cat-scratch disease, anthrax, tularemia, primary inoculation tuberculosis, atypical mycobacteria, syphilitic chancre, sporotrichosis, keratoacanthoma, and pyogenic granuloma [4].

A review of the literature on human orf infection reveals that some complications such as fever, lymphangitis, lymphadenopathy, and secondary bacterial infection have been noted. Very rare associations with papulovesicular eruptions have also been described, including a bullous pemphigoid-like eruption [4].

Although the primary site of infection is a relatively benign cutaneous lesion, the secondary complication of erythema multiforme has been surprisingly well described in relation to cases of orf, particularly in Europe [3].

The immune response to orf infection is considered to be responsible for erythema multiforme. Development of erythema multiforme following orf infection is very rare [4, 5].

The infection is usually clinically diagnosed on the basis of exposure history and the presence of a characteristic lesion. Virus isolation in tissue culture usually requires primary ovine or bovine cells and may be difficult to attain. The development of immunologic sera specific for parapoxviruses is another source for diagnostic reagents [1, 4].

Spontaneous resolution occurs in about 4 to 6 weeks. The disease is usually self-limiting and there is no specific treatment. For the treatment of uncomplicated orf infection, local antiseptic dressing is recommended in order to prevent secondary bacterial infections. Cidofovir cream and cryotherapy have recently been used successfully to treat very large lesions [1, 4, 6]. In our case, systemic steroid and hydroxyzine were used to treat erythema multiforme and bullous pemphigoid-like eruptions.

## 4. Conclusions

Since orf is a self-limited disease, early diagnosis is important to prevent complications and avoid improper treatment. Taking a precise and consistent history of the patient is a correct way to reach a diagnosis. We should know the rare complications of this disease such as erythema multiforme and bullous pemphigoid-like eruptions to perform proper interventions.

For prevention and control of infection, we should also identify the roots and routes of transmission and try to update and elevate the level of people's vision about this disease through outreach and education at the community. With knowing the disease may occur at any location, so all clinicians and researchers need to have this disease in mind as a differential diagnosis in patients who have a history of working or contact with animals.
